# Virtual Reality Technology in Nursing Professional Skills Training: Bibliometric Analysis

**DOI:** 10.2196/44766

**Published:** 2023-08-21

**Authors:** Chengang Hong, Liping Wang

**Affiliations:** 1 School of Nursing Hangzhou Normal University Hangzhou China

**Keywords:** virtual reality, VR, nursing professional skills, bibliometric analysis, visual content analysis, extended reality, XR, augmented reality, AR, mixed reality

## Abstract

**Background:**

Nursing professional skills training has undergone significant transformation due to the exponential growth of computer and medical technology. The innovative use of virtual reality (VR) in nursing education has emerged as a cutting-edge technical support technique that has gained attention as a highly effective method for improving nurse training quality.

**Objective:**

This study aims to review the current status of VR technology in nursing professional skills training, research hotspots, and emerging trends in the last 15 years.

**Methods:**

The Web of Science Core Collection database was used to search for literature on VR technology in nursing professional skills training covering the period from 2006 to 2022. Biblioshiny (K-Synth Srl) was used to import and convert the records to Bibliometrix (K-Synth Srl) for analysis, and R (R Core Team) was used for descriptive bibliometric analysis. VOSviewer (Leiden University) was used to cluster co-occurring keywords, and Scimago Graphica (version 1.0.16; Scimago Lab) was used to generate a geographical visualization of published countries and regions.

**Results:**

A total of 1073 papers were analyzed, indicating a surge in research on the application of VR in nursing professional skills training in recent years, as evidenced by a positive trend in annual publication of relevant literature. The majority of studies were from the United States (n=340) and Canada (n=107), and Margaret Verkuyl was the most prolific author, leading the way with 9 publications. Furthermore, “Computerized Virtual Patients in Health Professions Education: a Systematic Review and Meta-Analysis” was the most frequently cited reference. Keywords such as *education*, *simulation*, *skills*, *students*, and *care* were most commonly used by researchers.

**Conclusions:**

The bibliometric analysis provides a comprehensive overview of the use of VR in nursing professional skills training, indicating that VR-based training is an effective means of improving the skills and competencies of nursing students and professionals alike. The COVID-19 pandemic has reinforced the importance of developing VR-based distance education, despite challenges such as integrating virtual and real-world training and mitigating safety risks.

## Introduction

### Background

Nursing work holds a crucial position in health care. Nursing students, serving as a valuable pool of talent for the clinical nursing team, are essential figures in the advancement of nursing practice. They play a pivotal role in promoting the growth and development of the nursing profession and are a crucial component of the health care workforce [1]. Nursing educators face a significant challenge in cultivating the necessary expertise and skills among nursing students to provide effective patient care [2]. Considering the low efficiency of traditional nursing professional skills training methods, it can be challenging to motivate nursing students to engage with enthusiasm [3]. To ensure that nursing students possess sufficient expertise and are qualified to work in hospitals, many nursing educators use a variety of teaching strategies, including using VR, to enhance the quality of nursing professional skills training.

A virtual reality (VR) system can be defined as a highly interactive 3D digital media environment that generates a simulated reality and creates an immersive experience through the user’s senses of vision, hearing, and touch. Essentially, it provides users with the feeling of being present in a separate virtual environment. The characteristics of VR systems can be summarized into 3 pivotal elements: immersion, interactivity, and sensory perception [4]. VR technology has been extensively applied in nursing theory teaching, nursing skills training, and clinical nursing teaching, both domestically and internationally. Yang and Huang [5] found that traditional nursing skills teaching fails to replicate the actual clinical environment. Students often lack the opportunity to gain genuine sensory experience of nursing operations and may learn various nursing procedures in a conventional and generic manner. This may hinder their ability to effectively transition into clinical work in the early stages of their career. Chu et al [6] showed that a VR-based immersive experiential learning model could be implemented for teaching and that situational learning was achieved through virtual laboratory and simulation training. Gao and Yao [7] provided a new idea for a method to teach endoscopic retrograde cholangiopancreatography to nurses by combining a VR endoscopic simulation system with microteaching. Jung and Park [8] built a head-mounted VR platform to enable students to observe the process of intravenous catheter implantation by modeling the angiography room. These examples provide a compelling argument for the potential of VR technology in enhancing the professional skills of nurses and nursing students.

### Research Problem and Aim

The concept of “bibliometrics” was proposed by Pritchard in 1969, and it is defined as “the application of mathematical and statistical methods to books and other knowledge dissemination media” [9]. Bibliometrics offers a highly efficient tool for analyzing the progress of scientific research. It enables quantification of information on a specific research topic derived from online scientific citation databases, such as details on authors working in a field, publication numbers, and the distribution of research institutions [10]. Bibliometrics is a powerful tool that can assist in identifying significant literature in a research field and provide valuable data, such as keywords, institutions, country linkages, and distribution characteristics, in the form of a knowledge map. By quantifying the current state and future trends of the research topic, bibliometrics serves as an essential aid in comprehending the development of a particular field [11]. Generally, the greater the number of references included in a bibliometric method, the better we can comprehend the landscape of a research field [12]. A scoping review conducted by Yang and Huang [5] outlines the applications of VR technology in nursing professional skills training. There has been limited research on the scientific output of this field using bibliometric and visualization analyses. As a result, it is critical to comprehend the research status and hot spots of this field to aid in the advancement of nursing professional skills for nurses and nursing students. This study strives to offer a comprehensive analysis of the research field using the Web of Science (WOS) Core Collection as a basis and to serve as a valuable reference for future research.

## Methods

### Data Sources and Search Strategy

For this study, articles and reviews that were published in English between 2001 and 2021 were included, using the WOS Core Collection database as the primary data source: (1) the retrieval expression was constructed using a formula that used a Boolean logic operator: TS=(virtual reality OR VR OR virtual medicine OR augment reality OR mixed reality OR virtual simulation) AND TS =(nursing* OR nursing practice OR nursing training OR academic training OR serious game), and the scope of literature was restricted to only articles and reviews; (2) the English language was selected; (3) there were 1073 documents retrieved in the search of WOS (as updated on 11 November 2022); and (4) all records were exported to plain text files, including the full record and cited references.

### Bibliometric Analysis

**Figure 1:**
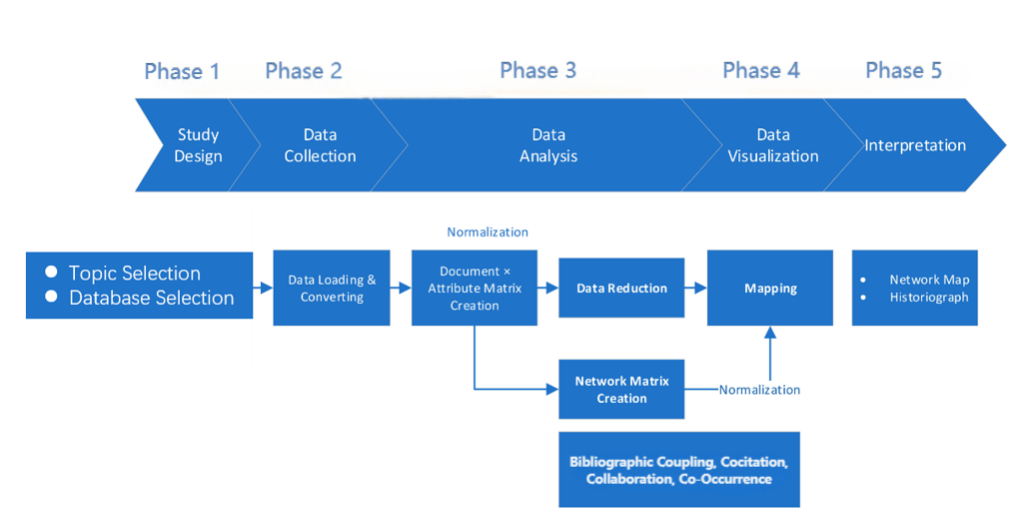
Diagram illustrating the methodology of the bibliometric analysis, adapted with consent from the work of Aria and Cuccurullo [13] and Secinaro et al [14].

A description of the bibliometric analysis method can be found in Aria and Cuccurullo [13]. Five rigorous steps, namely study design, data collection, data analysis, data visualization, and interpretation, were included in the analysis [14]. The complete methodology is schematically illustrated in Figure 1. The initial stage was the study design phase; nursing professional skills training was selected as the study topic. The research data resource chosen was the WOS Core Collection database, from which literature retrieval returned 1242 documents during the data collection phase. The review followed the structured approach outlined by Kable et al [15] for researching and evaluating literature. In this study, 2 reviewers (CH and LW) conducted an independent screening of the search results, with cross-checking used to resolve any disagreements. Training in document retrieval and screening was received by all researchers based on the textbook *Medical Literature Information Retrieval* [13]. A document-type filter was applied to the WOS, and articles and data papers were included, resulting in a sample of 1073 papers published between 2006 and 2022. All records were imported to Biblioshiny (K-Synth Srl) and converted to Bibliometrix (K-Synth Srl) for subsequent analysis. In the third stage, R (R Core Team) was used for descriptive bibliometric analysis and to develop a matrix of all documents. Biblioshiny, the Tidyverse package ggplot2 (Hadley Wickham), and VOSviewer (Leiden University) were used in the fourth stage to create a keyword cluster map. Scimago Graphica (version 1.0.16; Scimago Lab) was used to generate geographic visualization maps of the countries or regions. To identify influential sources, the Bradford law was used to unveil the journal distribution. The Methods section presents an interpretation of the data analysis and visualization outcomes.

### Ethical Considerations

An ethical approval application was not filed for this paper. The Chinese Hospital Association has stated that an ethics review is unnecessary for secondary analyses of published data.

## Results

### Descriptive Bibliometric Analysis

**Figure 2:**
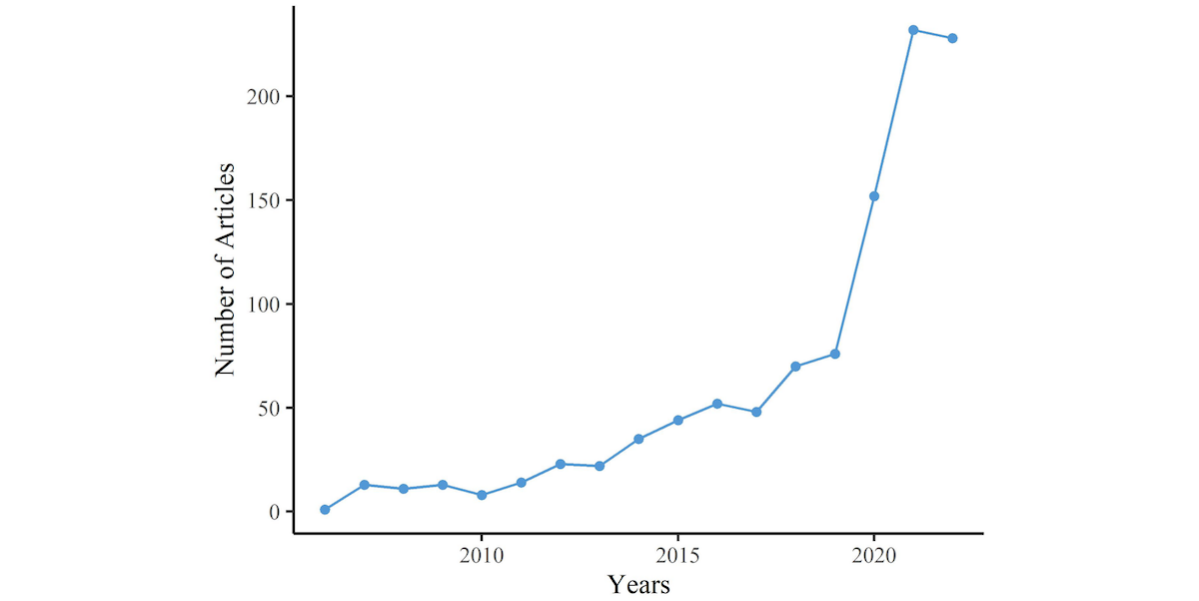
The production of research on virtual reality–based nursing professional skills training in the scientific domain between 2006 and 2022.

Each period’s publication count is a reflection of the research field’s development trends. Figure 2 presents the scientific output over the course of the study. There were 3 periods based on the number of publications: 2006 to 2013, 2014 to 2019, and 2019 to 2022. As seen in Figure 2, no relevant papers were released before 2006. From 2006 to 2013, the number of published articles increased annually, but at a relatively sluggish pace. The quantity of publications in 2019 (n=76) surpassed 75 for the first time. The number of articles published between 2019 and 2021 increased significantly, indicating that more scholars were focusing on the potential of VR in the nursing professional skills training field. Table 1 provides essential details on the 1073 research papers published in the WOS Core Collection database from 2006 to 2022. The annual average number of published research papers was 61.29, with each paper having an average citation count of 11.52. These papers had 4369 authors, with 59 of them being single-author papers. The average number of authors for each research paper was 2 (2.33). The collaboration index, which is the total number of authors of multi-authored papers divided by the total number of multi-authored papers, was 2.38. These papers also produced a total of 2388 author keywords.

**Table 1:** Crucial information on virtual reality–based nursing professional skills training literature determined by the bibliometric analysis.

Information	Values
Documents, n	1073
Sources^a^ (frequency distribution), n	300
Timespan (years)	2006-2022
References, n	30,766
Author keywords, n	2388
Keywords Plus^b^, n	1469
Authors, n	4369
Authors’ appearances (frequency distribution), n	5283
Authors of single-authored documents, n	59
Authors of multi-authored documents, n	1014
Documents per author (average), n	0.246
Coauthors per document (average), n	4.92
Citations per document (average), n	11.52
Collaboration index	0.23

^a^Sources include journals and books.

^b^Total number of phrases that frequently appear in the title of an article’s references.

### The WOS Research Areas

**Figure 3:**
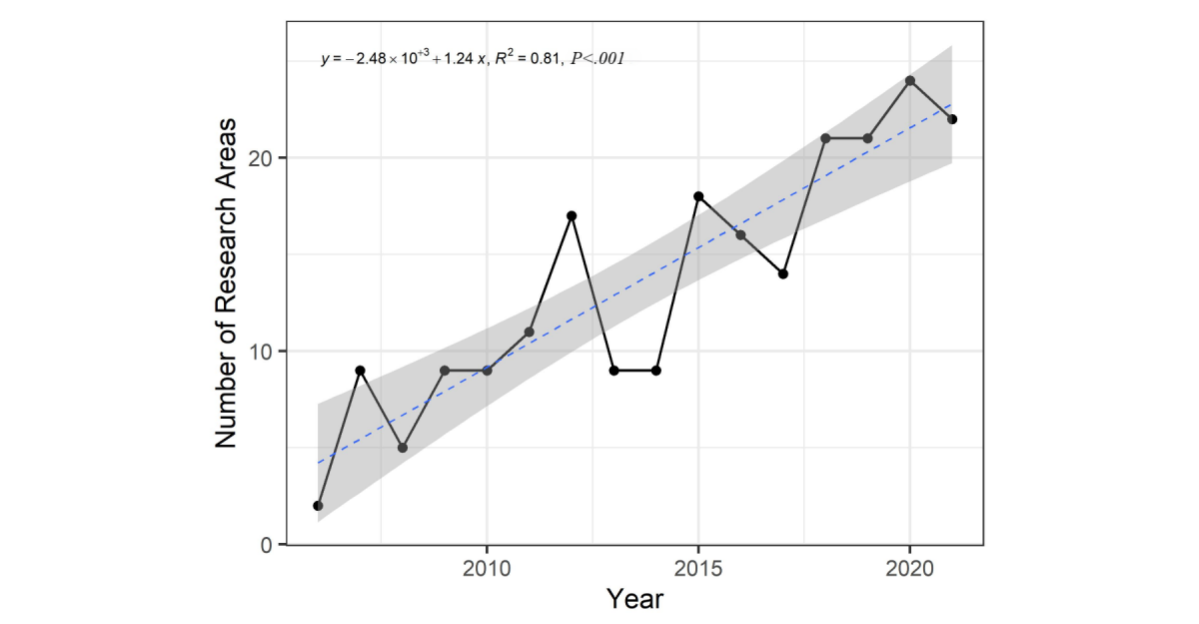
The count of Web of Science research areas included in the literature of VR-based nursing professional skills training.

**Figure 4:**
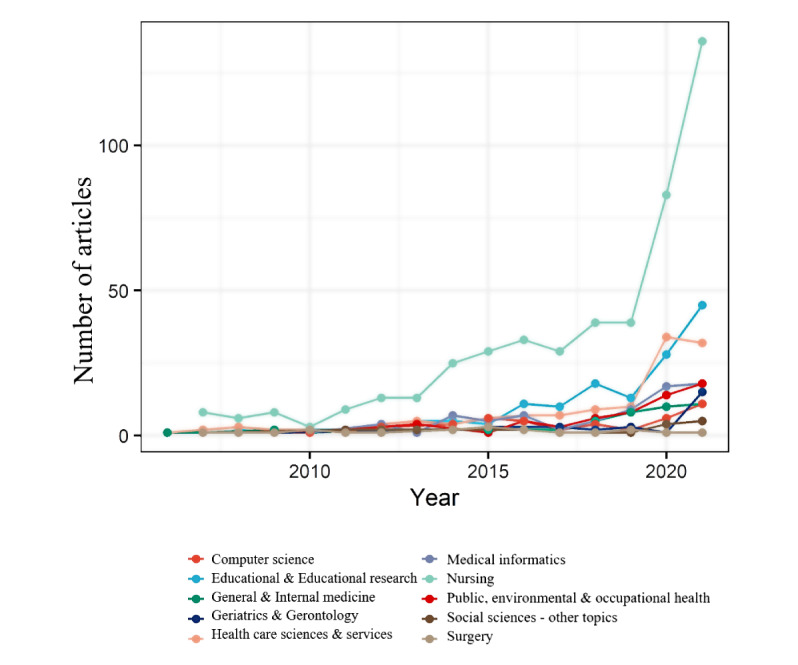
Yearly variations in the productivity of the top 10 Web of Science research areas in specific fields.

One or more subject categories (SCs) are linked to each publication indexed in the WOS. In this study, the number of research areas covered by VR-based nursing professional skills training education literature increased from 2 in 2006 to 22 in 2022 (Figure 3). Figure 4 displays the annual changes in productivity in the top 10 areas of VR-based nursing professional skills training education, which illustrates changes in the focus areas of VR-based nursing professional skills training education. The most popular research area was nursing, followed by education and educational research and health care sciences and services. With the increasing maturity of VR technology and stable production of VR equipment, the explosive growth in publications in the field of nursing professional skills training can be attributed to the use of various teaching strategies, including VR, by many nursing educators to enhance the quality of education.

### Research Countries and Institutions

**Figure 5:**
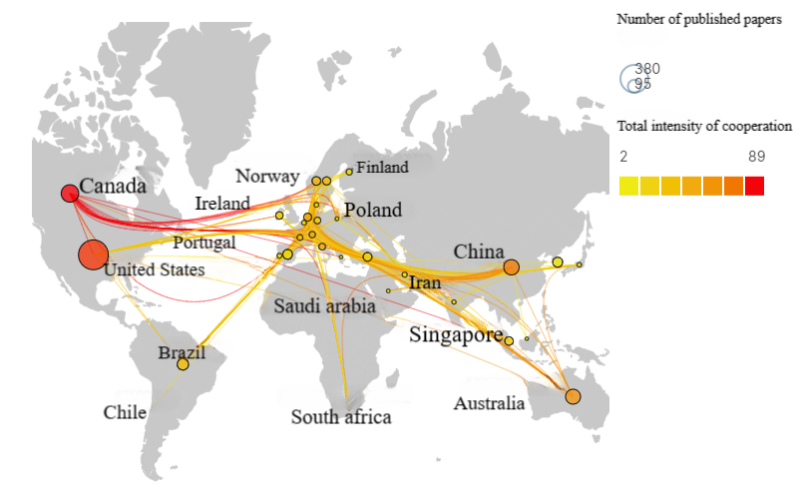
Map displaying research collaboration between countries.

Table 2 highlights that the United States has contributed the highest number of publications (340/1042, 32.63%) in the domain of VR-based nursing professional skills training, followed by Canada (107/1042, 10.27%), China (89/1042, 8.54%), Australia (71/1042, 6.81%), and the United Kingdom (51/1042, 4.89%). Of the total number of articles, 64.65% (658/1042) were published in these 5 countries. To generate a geographic visualization map (Figure 5), countries with more than 5 publications were analyzed using Scimago Graphica. The lines on the map indicate the degree of cooperation between different countries, with the thickness of the lines reflecting the extent of cooperation. It is evident from the map that the United States, Canada, Australia, China, and other countries have actively collaborated with other countries. Additionally, several scholars from Europe have shown interest in VR-based nursing professional skills training education and have conducted joint research projects to a certain extent. Table 3 highlights that while the United States has the highest number of publications, Canada’s top 10 institutions rank higher in total citations, suggesting that both countries have robust support and funding for VR-based nursing professional skills training research.

**Table 2:** Countries with the highest total citation count for virtual reality–based nursing professional skills training research, ranked by the top 10.

Ranking	Country	Articles, n	Citations, n
1	United States	340	4215
2	Canada	107	1186
3	China	89	867
4	Australia	71	1145
5	United Kingdom	51	1027
6	Brazil	49	227
7	Korea	42	181
8	Spain	35	309
9	Singapore	30	489
10	Turkey	28	115

**Table 3:** The institutions ranked in the top 10 based on the total number of citations for virtual reality–based nursing professional skills training.

Institution	Country	Articles, n
University of Toronto	Canada	42
National University of Singapore	Singapore	38
Université de Montréal	Canada	23
Toronto Metropolitan University	Canada	22
Centennial College	Canada	20
Johns Hopkins University	USA	20
University of Ottawa	Canada	19
University of São Paulo	Brazil	19
Nanyang Technological University	Singapore	18
University of Almeria	Spain	18

### Most Influential Source Journals

**Figure 6:**
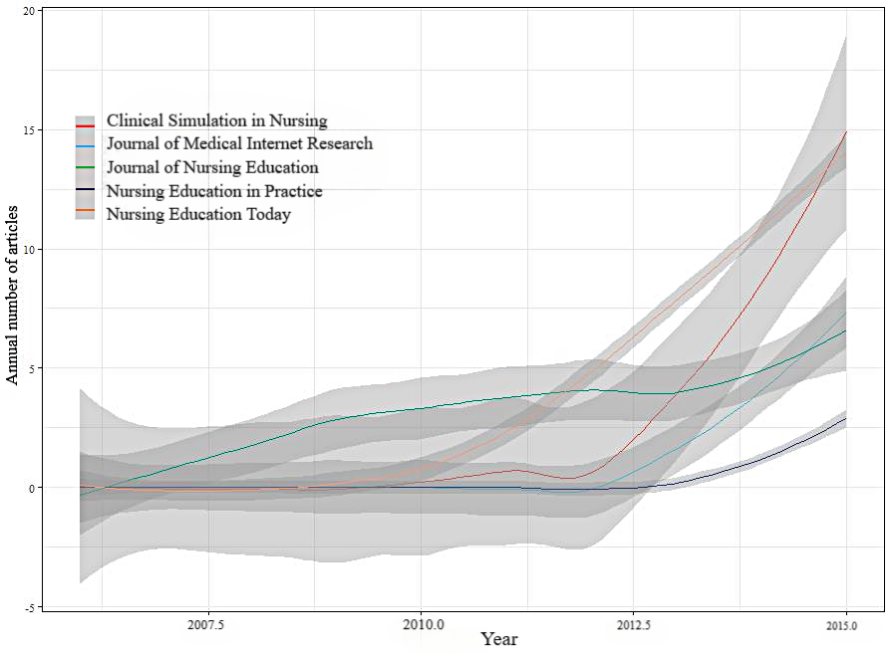
Temporal analysis of the publication sources of virtual reality–based nursing professional skills training.

Table 4 shows that *Clinical Simulation in Nursing* was the most cited journal with 1401 of 30,766 total citations (4.55%), while the *Journal of Medical Internet Research*, with an impact factor of 7.077, was the most impactful among the top 10 academic journals. Half of the journals in the table were ranked in the top quartile position. We also analyzed the distribution of research papers among leading sources and found that the top 5 journals accounted for 304 of 1042 (29.17%) of the total number of papers. The top 5 journals with the highest numbers of papers published were *Clinical Simulation in Nursing* (n=125), *Nurse Education Today* (n=93), *Journal of Nurse Education* (n=32), *Journal of Medical Internet Research* (n=27), and *Nurse Education in Practice* (n=27), with *Clinical Simulation in Nursing* having the most local citations (Table 4). According to the Bradford law, the source journals of VR-based nursing professional skills training papers were widely scattered, and the top 10 most influential journals were selected based on the number of local citations. Journals that were identified as core source journals in the field of VR-based nursing professional skills training according to the Bradford law are marked in Figure 6. These included *Clinical Simulation in Nursing*, *Nurse Education Today*, *Journal of Nurse Education*, *Journal of Medical Internet Research*, *Nurse Education in Practice*, *CIN-Computers Informatics Nursing*, and *Journal of Advanced Nursing*. These journals played a crucial role in the development of VR-based nursing professional skills training during the study period.

**Table 4:** Ranking of the top 10 journals in VR-based nursing professional skills training based on the number of local citations.

Journal	Local citations, n	Documents, n	Impact factor^b^	Rank (quartile)
*Clinical Simulation in Nursing* ^a^	1401	125	2.856	Q1
*Nurse Education Today* ^a^	1397	93	3.906	Q1
*Journal of Nursing Education* ^a^	526	32	2.381	Q2
*Journal of Medical Internet Research* ^a^	470	27	7.077	Q1
*Nurse Education In Practice* ^a^	440	27	3.43	Q1
*CIN-Computers Informatics Nursing* ^a^	421	25	2.146	Q3
*Journal of Advanced Nursing* ^a^	407	19	3.057	Q1
*Nurse Educator*	385	18	2.791	Q1
*JMIR Serious Games*	382	18	3.364	Q2
*Nursing Education Perspectives*	343	17	0.59	Q3

^a^Journals classified as core resources in virtual reality–based nursing professional skills training according to the Bradford law.

^b^Impact factor in 2021.

### Most Influential Authors

**Figure 7:**
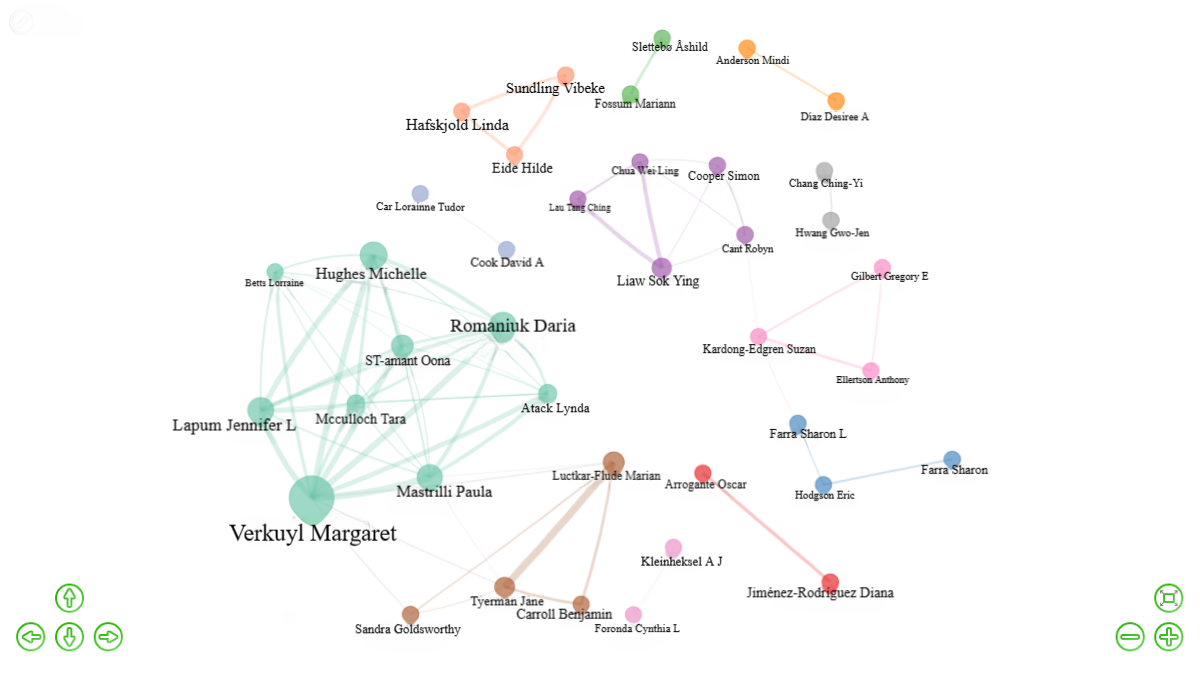
Visualization map of author collaboration in research on virtual reality–based nursing professional skill training.

The H index is a widely accepted measure of scientific performance based on the number of citations received by a scientist’s published papers [16]. Table 5 shows the top 10 authors with the largest H index: Margaret Verkuyl (H index 9), Shujuan Liao (H index 7), Daria Romaniuk (H index 7), Hilde Eide (H index 6), Sharon L Farra (H index 6), Michelle Hughes (H index 6), Paula Mastrilli (H index 6), Michelle Aebersold (H index 5), Lynda Atack (H index 5), and Luciana Mara Monti Fonseca (H index 5). The researcher with the highest number of citations, indicating significant influence, was Margaret Verkuyl. She is also the author with the highest density of collaboration (Figure 7). The top 10 most influential researchers included researchers from Canada (n=5), China (n=1), Norway (n=1), Brazil (n=1), and the United States (n=2). The 1073 papers involved 4369 authors. In total, 59 authors published 65 documents as sole authors, with an average of 4.92 co-authors per document and a collaboration index of 0.23. Each author contributed an average of 0.246 documents to the field, and on average, each document had 1.1 authors and 4.92 coauthors. These findings suggest that VR-based nursing professional skills training research is often a collaborative effort involving multiple authors.

**Table 5:** Authors ranked by the H index, with the top 10 being the most influential.

Author	H Index	G Index	Times cited^a^, n	Scientific productions, n	First year published	Country
Margaret Verkuyl	9	16	268	21	2016	Canada
Shujuan Liao	7	13	250	13	2014	China
Daria Romaniuk	7	9	183	9	2016	Canada
Hilde Eide	6	10	124	10	2011	Norway
Sharon L Farra	6	7	114	7	2013	United States
Michelle Hughes	6	9	147	9	2017	Canada
Paula Mastrilli	6	10	165	10	2016	Canada
Michelle Aebersold	5	5	126	5	2015	United States
Lynda Atack	5	7	137	7	2016	Canada
Luciana Mara Monti Fonseca	5	8	68	8	2014	Brazil

^a^Web of Science Core Collection times cited count.

### Most Influential Papers

This section provides a list of the most influential papers (based on the number of citations) published between 2006 and 2022. Cocitations within a group of authors suggest that certain documents may include concept symbols. It is noteworthy that Tables 6 and 7 show a difference between the local citation score (LCS), which reflects the number of citations within the field, and the global citation score (GCS), which represents the total number of citations in WOS. The paper with greatest influence according to LCS was “Clinical Virtual Simulation in Nursing Education: Randomized Controlled Trial” (n=49). The paper with greatest influence according to GCS was “Computerized Virtual Patients in Health Professions Education: a Systematic Review and Meta-Analysis” (n=273). The papers in question may encompass theories that form the foundation of the field, early works that lay groundwork, and methodological principles [17].

**Table 6:** Top 10 papers ranked by the local citation score.

Paper	Year	Journal	Local citation score	Global citation score
Padilha et al [18]	2019	*J Med Internet Res*	49	108
Foronda et al [19]	2020	*Simul Healthc*	49	87
Cobbett and Snelgrove-Clarke [20]	2016	*Nurs Educ Today*	43	62
Cant and Cooper [21]	2014	*Nurs Educ Today*	39	94
Butt et al [22]	2018	*Clin Simul Nurs*	35	74
Cook et al [23]	2010	*Acad Med*	33	273
Padilha et al [24]	2018	*Clin Simul Nurs*	27	48
Duff et al [25]	2016	*Clin Simul Nurs*	26	50
Verkuyl et al [26]	2017	*Clin Simul Nurs*	26	50
Kidd et al [27]	2012	*J Psychosoc Nurs Men*	26	60

**Table 7:** Top 10 papers ranked by the global citation score.

Paper	Year	Journal	Local citation score	Global citation score
Cook et al [23]	2010	*Acad Med*	33	273
Cook et al [28]	2013	*Acad Med*	3	193
Zendejas et al [29]	2013	*J Gen Intern Med*	1	173
Foronda et al [30]	2016	*Nurse Educ Pract*	3	166
Gentry et al [31]	2019	*J Med Internet Res*	22	135
Kipping et al [32]	2012	*Burns*	6	130
Triola et al [33]	2006	*J Gen Intern Med*	13	123
Padilha et al [18]	2019	*J Med Internet Res*	49	108
Robert et al [34]	2014	*Front Aging Neurosci*	1	107
Manera et al [35]	2015	*Front Aging Neurosci*	3	106

### Distribution of Keywords

**Figure 8:**
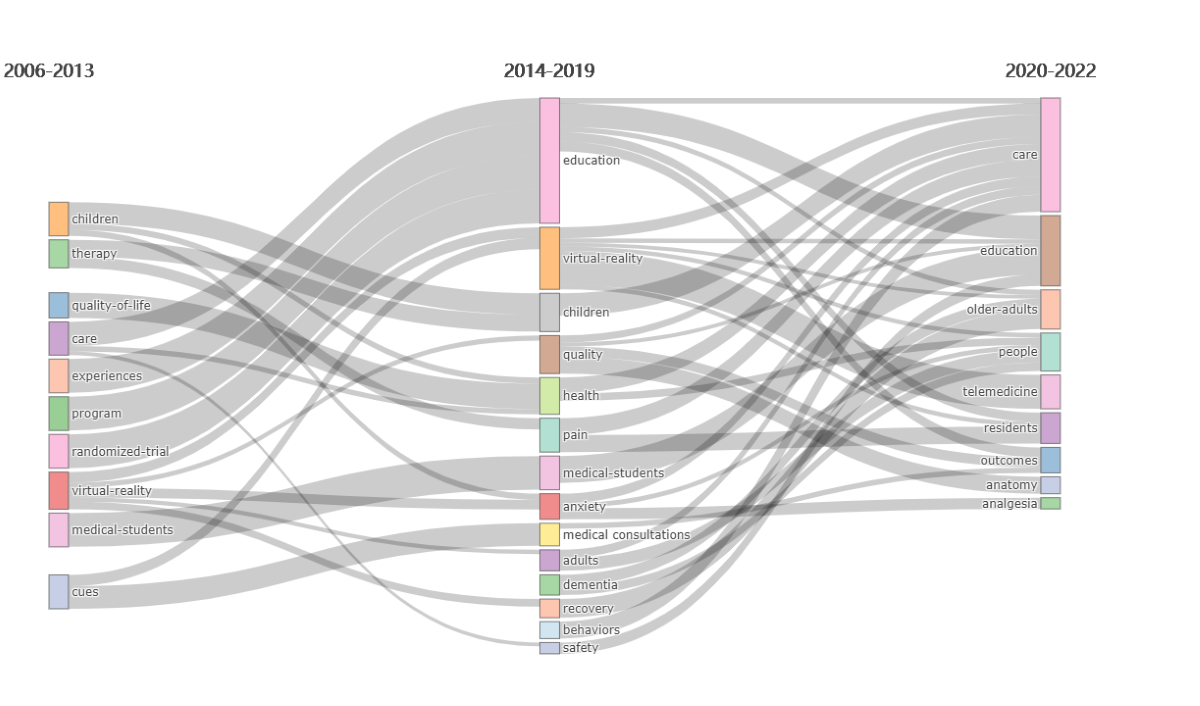
Map showing thematic evolution of keywords.

By conducting data cleaning to eliminate coding errors that could arise from different articles using varying keywords to introduce the same concept, we identified 2388 author keywords in 1073 published papers on VR-based nursing professional skills training between 2006 and 2022. The keyword analysis enables summary of the study’s topics in the field and exploration of hotspots. For instance, *nursing* and *nurses* were recorded as *Nurses* to prevent discrepancies. *Education* had the highest frequency of 178 among the keywords, with other high-frequency keywords being *Simulation* (n=148), *Skills* (n=95), *Students* (n=84), *Care* (n=77), *Impact* (n=64), *Knowledge* (n=64), *Nurses* (n=62), *Virtual-reality* (n=58), and *Technology* (n=47), as shown in Table 8. Additionally, Multimedia Appendix 1 shows a keyword cluster map created using VOSview, while Figure 8 depicts the thematic evolution of the keywords.

**Table 8:** Top 10 keywords related to the application of virtual reality in nursing professional skills training.

Ranking	Keyword	Frequency	Centrality
1	*Education*	178	0.06
2	*Simulation*	148	0.05
3	*Skills*	95	0.04
4	*Students*	84	0.03
5	*Care*	77	0.05
6	*Impact*	64	0.06
7	*Knowledge*	64	0.05
8	*Nurses*	62	0.02
9	*Virtual-reality*	58	0.03
10	*Technology*	47	0.05

## Discussion

### General Information

Based on the annual number of publications, the publication years can be divided into 3 phases: 2006 to 2013, 2014 to 2019, and 2019 to 2022. The first phase, spanning from 2006 to 2013, saw an increase in the number of articles, albeit at a relatively slow growth rate. The second phase, from 2014 to 2019, was marked by significant advancements in VR device and software technology. This was exemplified by the launches of Project Glass by Google in 2012 and the first generation of the Oculus Rift VR headset by Oculus. These improvements resulted in an increase in the availability and quality of VR components [17]. In the period between 2014 and 2021, there was a noticeable increase in interest among scholars in computer science, as evidenced by periods 2 and 3. In the most recent phase, from 2019 to 2021, the number of publications exceeded 75 for the first time, with 76 articles published. This trend indicates an expanding interest among scholars in exploring the potential of VR technology in the field of nursing professional skills training.

Contributions to VR-based nursing professional skills training came from various countries worldwide, including the United States, Canada, China, Australia, the United Kingdom, Brazil, Korea, Spain, Singapore, and Turkey, with some taking a more prominent role. Notably, China is one of the few Asian countries to contribute to this field, indicating a lack of research emphasis on this topic among Asian scholars. In contrast, Canadian institutions and scholars have demonstrated continued interest in research in this area. A bibliometric analysis of VR and nursing professional skills training revealed Canada to have the highest contribution among all countries [23].

The frequency of cocitation can serve as a reflection of a journal’s quality and influence. Among the ten journals with the highest cocitation frequency, half were found to be ranked in the top quartile. This suggests significant impact and recognition in the international community, indicating that the application of VR in nursing professional skills training has gained worldwide attention.

Following the analysis of cocited papers, it was found that “Clinical Virtual Simulation in Nursing Education: Randomized Controlled Trial” (n=49) was the most influential paper in terms of LCS. This study may contain foundational theories, pioneering early work, and methodological principles in the field. Similarly, “Computerized Virtual Patients in Health Professions Education: a Systematic Review and Meta-Analysis” (n=273) was deemed the most influential paper in terms of GCS. These works demonstrate how innovative research can impact an academic discipline for years to come and continue to inform contemporary research in the field.

### Research Hot Spots and Frontiers

By using a combination of high-frequency keyword statistics, visualization analysis, and including relevant literature, this study indicates that an overwhelming majority of both domestic and international researchers have concentrated their efforts on exploring the application of VR technology in nursing education with a particular emphasis on nursing professional skills training.

Owing to variations in technological advancement, each country and region has conducted research to its own specifications with regards to the use of VR in nursing education. As an illustration, the VR nursing skills training system can provide a significant contribution to ensuring the proficiency of nursing students by enabling them to learn essential operational skills without the need for physical attendance, thereby providing them with the opportunity to review training materials anywhere at any time [23]. International research in this field commonly uses desktop VR system software to enhance students’ clinical thinking skills. This type of software emphasizes immersive patient scenarios presented on the computer screen in which students are “placed” in a simulated clinical setting to observe and address the patient’s discomfort and pain. The aim is to develop students’ proficiency in making informed clinical decisions based on complex patient conditions [36]. The VR system can be improved in a short time according to teaching and learning needs, quickly meeting the skills training and teaching needs of various courses and improving the training effect. In order to give full play to the maximum benefit of the virtual system, it is suggested that schools and hospitals with suitable conditions choose VR technology to carry out skills training for students and promote their early adaptation to clinical practice.

In the field of nursing education, there are notable technical issues that exist with the application of VR technology at present. Caylor et al [37] pointed out that VR systems are faced with the problem of unclear navigation mechanisms within the operation interface. Poor experiences among nursing students during simulation learning are attributed to the confusing nature of the navigation interface within VR software platforms. Wilson et al [38] suggested that in future research on VR technology more attention be given to creating a simulation system with high fidelity, increasing the complexity of the cases presented within VR and reproducing challenging clinical case scenarios to enhance subjects’ diagnostic reasoning abilities. Moreover, the development level and quality of VR software must be improved. This improvement is necessary due to the lack of understanding among VR technicians of the medical care field, which leads to failures to accurately reflect the key points and difficulties that teachers aim to emphasize. Additionally, it is vital to combine the advantages of VR teaching with existing nursing practices to ensure that VR technology is used effectively in nursing education [39]. To maximize the potential of VR technology in nursing education, researchers must consider providing a more comprehensive VR technology intervention, emphasizing multidisciplinary cooperation, and enhancing the familiarity of software technology developers with the nursing field. Additionally, teachers’ proficiency in the operation of the virtual teaching process must be improved. To better explore the efficacy of VR technology in nursing education, it is crucial to enhance the VR teaching environment to improve nursing students’ ability to apply the skills learned in virtual settings to clinical practice.

### Applications and Development Trends in Research

Over the last 15 years, VR technology has been extensively implemented in nursing professional skills training. As mobile computing and sophisticated software programs continue to develop, highly immersive VR systems have become increasingly affordable and accessible [40]. Popular VR systems currently used in nursing professional skills training include Kinect from the United States, Nintendo Wii from Japan, HTC Vive from Taiwan, China, and Samsung Gear VR from South Korea. By integrating these VR systems into nursing education, we aim to facilitate the development of students’ initiative and promote self-directed learning abilities, ultimately enhancing their clinical practice acumen.

As COVID-19 continues to impact the world, students’ access to skills training may be limited. However, VR technology offers a viable solution to this problem. VR-based nursing skills training immerses students in a simulated environment, offering an opportunity to develop critical skills despite the pandemic. According to research, the use of a VR immersive experiential learning model for teaching fosters situational teaching through virtual laboratory and simulation training [6]. Furthermore, Gao and Yao [7] combined use of a VR endoscopic simulation system with the microteaching method to provide a new idea for teaching nurses endoscopic retrograde cholangiopancreatography. Jung and Park [8] developed a head-mounted VR platform for observing the process of intravenous catheter implantation in a highly realistic sensory scenario. This platform enabled students to view the entire arterial and venous system of the body, strengthening their theoretical and practical operation skills. Zhang et al [41] developed a highly realistic scenario simulation system for improving teachers’ teaching ability and cultivating students’ initiative, enthusiasm, and autonomous learning ability.

To establish VR technology as a reliable tool for improving nursing professional skills training, researchers must integrate knowledge from various fields, including nursing, computer science, neuroscience, psychology, social cognition, multisensory perception, and multimedia development. This requires an interdisciplinary approach that takes into account the needs of all stakeholders, including students, teachers, nurses, and software developers [42]. Thus, research on VR-based nursing professional skills training must involve a broad range of individuals to create effective teaching strategies that meet the unique needs of every student. High-quality research is necessary to explore the full potential of VR technology across various disciplines.

### Strengths and Limitations

This is the first bibliometric analysis of VR-based professional skills training in nursing, providing a comprehensive understanding of the current state of VR in nursing education. Through analyzing keywords, countries or regions, institutions, authors, journals, and references, we have gained valuable insight into the research landscape. In the future, longitudinal in-depth studies on current hot topics or large, multicenter, high-quality randomized controlled studies guided by research trends can further enhance this field. At present, research in this field is predominantly limited due to technology and cost. To promote growth, policy and financial support should be provided to encourage researchers to learn from foreign advanced experience and enhance VR technology. Nursing staff must participate in program design from a nursing education perspective to create more suitable VR equipment and procedures. By vigorously developing the application of VR in the field of nursing education, we can better prepare our students for clinical practice.

However, it is important to note that this study used only one database (WOS), which may have excluded high-quality papers in the field. Additionally, the current word segmentation algorithm used in bibliometrics lacks necessary intelligence, leading to inaccurate extraction of some keywords. Thus, future bibliometric studies must strive to enhance the semantic understanding of citation data to facilitate accurate word segmentation and intelligent extraction of bibliometric knowledge. To ensure a more comprehensive and precise theoretical basis, future research should expand the database and source high-quality papers both domestically and internationally. This will better promote the advancement of nursing education and practice to new levels.

### Conclusions

To review the application of VR technology in nursing professional skills training, this study used the WOS database, R, Scimago Graphica, and VOSview to provide a scientific and intuitive analysis. Bibliometric analysis indicates that research on VR-based professional skills training in nursing has experienced rapid development over the past 15 years. This is evident from the rising number of publications in core journals and increased collaboration between authors and countries or regions. Although this paper demonstrates an exponential growth trend in VR-based nursing professional skills training and positive feedback in the field’s advancement, further investigation is required to understand the feedback mechanism.

## Appendix

Multimedia Appendix 1 Map showing research collaboration between keywords.

## Abbreviations

GCS: global citation score
LCS: local citation score
SC: subject category
VR: virtual reality
WOS: Web of Science
