# Supplementary figures and images for: Virtual Reality Technology in Nursing Professional Skills Training: Bibliometric Analysis
Source: JMIR Serious Games. 2023 Aug 21;11:e44766. doi: 10.2196/44766 (PMC10477921; doi:10.2196/44766)

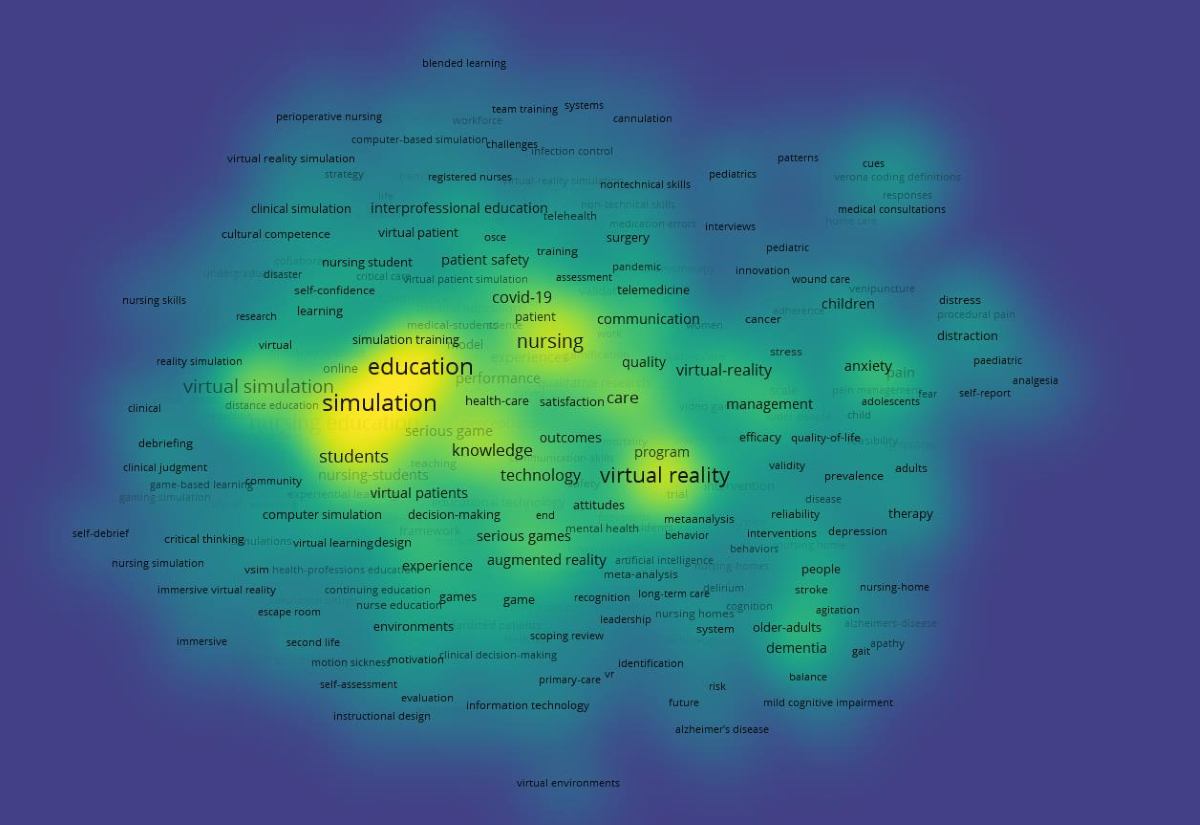

Supplement: Multimedia Appendix 1 [file games_v11i1e44766_app1.png]
